# Virus Isolation and “Acute” West Nile Virus Encephalitis (Response to Huang et al.)

**DOI:** 10.3201/eid0905.030018

**Published:** 2003-05

**Authors:** Vijay K Krishnamoorthy, Jayashri Bhaskar, John N Sheagren

**Affiliations:** * Advocate Illinois Masonic Medical Center, Chicago, Illinois, USA

**To the Editor:** We read with interest a recent article in your journal, First Isolation of West Nile virus from a Patient with Encephalitis in the United States (*1*); in the report, we were unable to ascertain indisputable evidence that this patient had indeed acquired acute West Nile virus (WNV) encephalitis. In animals (*2*,*3*) and humans (*4*), West Nile virus can persist in the host even after the host has recovered from an acute WNV infection, presumably more so in the immunocompromised persons. Therefore, in the case described by Huang et al. (*1*), proving that the patient did not have a history of WNV infection is important, particularly because this pa

tient is from a geographic area where WNV is known to exist. The findings at autopsy of perivascular lymphocyte cuffing in mammillary bodies of the brain are not the classic findings reported during the West Nile encephalitis outbreak in New York City (*5*). The immunoglobulin (Ig) G antibody against WNV, if it had been present, would have been useful in that IgG antibody in the absence of IgM antibody is indicative of past rather than acute infection.

The WNV copy numbers in clinical samples and clinical indices (leukocyte count) suggest that the virus multiplies in the setting of leukopoenia or immune suppression and cannot be definitive proof that it was an acute infection, unless a negative preillness sample was available. The cause of the transient viremia, whether acutely acquired or from increased proliferation in a chronic infection, needs to be clarified further. In the future, antigen detection will guide patient management decisions; therefore, the possibility of a human chronic carrier state warrants study.
